# Comparative analysis of the impact of psychosocial stress on exacerbation and dynamic of psychosomatic pathology (on the model of psoriasis) in periods of large-scale social upheavals (Covid-19 and a large-scale war in Ukraine)

**DOI:** 10.1192/j.eurpsy.2025.593

**Published:** 2025-08-26

**Authors:** M. Markova, H. Prib, A. Markov, R. Yaremkevich

**Affiliations:** 1 Sexology, Psychotherapy and Medical Psychology, Kharkiv National Medical University, Kharkiv; 2 Psychology, Academy of Labour, Social Relations and Tourism, Kyiv; 3 Skin and Venereal Diseases, Uzhgorod National University, Uzhgorod, Ukraine

## Abstract

**Introduction:**

The large-scale social stress of recent years (the COVID-19 pandemic, the large-scale invasion of the rf in Ukraine) has a powerful negative effect on the psyche of people and causes an increase in the incidence of stress-associated pathology. One of the vulnerable groups in the formation of distress conditions is patients with psychosomatic pathology, in particular, psoriasis, in the pathogenesis of which psychosocial stress plays an important role.

**Objectives:**

To determine the existence of a differentiated impact of a contain of the large-scale stressful events on the health of patients with psychosomatic pathology.

**Methods:**

83 psoriasis patients interactively were examined using the Google Form developed by us, which provided for a structured interview with the purpose of determining anamnestic data and collecting subjective complaints of the patient in two time periods the year of quarantine due the Covid-19 pandemic (11.03.2020-11.03.2021), and the year from the beginning of full-scale russian aggression (24.02.2022-24.02.2023). Statistical analysis included descriptive statistics and assessment of the significance level of differences using Fisher’s exact test (one-sided).

**Results:**

In the 1st year of the Covid-19 pandemic, exacerbation of psoriasis was found in 36.1% of patients, of which 63.3% believed that this was associated with the

impact of stress through pandemic. The stress of war proved to be a more powerful psycho-traumatic factor (P<0.01): in the 1st year, exacerbation of psoriasis was observed in 61.4% of patients, 88.2% of whom associated it with the impact of war. The prevalence of almost all psychopathological symptoms in patients during a full-scale war was slightly higher than during the Covid-19 pandemic: fear and anxiety (80.0% VS 88.2%), reduced mood (66.7% VS 90.2%, P<0.01), dyssomnia (50% VS 45.1%), asthenia (36.7% VS 41.3%), emotional lability (33.3% VS 37.3 %).

**Conclusions:**

The conducted study revealed the impact of strong social stress on the frequency of psoriasis exacerbations and mental maladjustment. The stress of the war turned out to be a more powerful stress factor, and the patients significantly more often associated this social stress, with the exacerbation of psoriasis. It is allows us to consider the content of the stress load and the peculiarities of individual perception of stress as a relevant factor in the exacerbation of psoriasis and comorbid mental disorders. But a larger study involving a larger number of patients is needed to reveal more subtle patterns.

**Disclosure of Interest:**

None Declared

